# Adequate enrollment of women in cardiovascular drug trials and the need for sex-specific assessment and reporting

**DOI:** 10.1016/j.ahjo.2022.100155

**Published:** 2022-06-23

**Authors:** Corinne Carland, Barinder Hansra, Cody Parsons, Radmila Lyubarova, Abha Khandelwal

**Affiliations:** a Department of Medicine, University of Pennsylvania, United States of America; b Division of Cardiology and Department of Critical Care Medicine, UPMC, United States of America; c Cardiovascular Health, Stanford Health Care, United States of America; d Division of Cardiology, Albany Medical College, United States of America; e Division of Cardiology, Stanford School of Medicine, United States of America

**Keywords:** Sex differences, Women's cardiovascular disease, Pharmacology, Clinical trials, Female, Disparities

## Abstract

Cardiovascular disease (CVD) is the leading cause of death for women in the United States and globally. There is an abundance of evidence-based trials evaluating the efficacy of drug therapies to reduce morbidity and mortality in CVD. Additionally, there are well-established influences of sex, through a variety of mechanisms, on pharmacologic treatments in CVD. Despite this, the majority of drug trials are not powered to evaluate sex-specific outcomes, and much of the data that exists is gathered post hoc and through meta-analysis. The FDA established a committee in 1993 to increase the enrollment of women in clinical trials to improve this situation. Several authors, reviewing committees, and professional societies have highlighted the importance of sex-specific analysis and reporting. Despite these statements, there has not been a major improvement in representation or reporting. There are ongoing efforts to assess trial design, female representation on steering committees, and clinical trial processes to improve the representation of women.

This review will describe the pharmacologic basis for the need for sex-specific assessment of cardiovascular drug therapies. It will also review the sex-specific reporting of landmark drug trials in hypertension, coronary artery disease (CAD), hyperlipidemia, and heart failure (HF). In reporting enrollment of women, several therapeutic areas like antihypertensives and newer anticoagulation trials fare better than therapeutics for HF and acute coronary syndromes. Further, drug trials and cardiometabolic or lifestyle intervention trials had a higher percentage of female participants than the device or procedural trials.

## 1 Introduction

Cardiovascular Disease (CVD) is the leading cause of death in men and women worldwide. As pharmacologic options for treating CVD and risk factors expands rapidly, there is a growing recognition of the underrepresentation of women in clinical trials. Concurrently, there is increasing evidence of sex-based differences in biology, pharmacology, and pharmacogenomics, which would impact prescribing and dosing of these medications for women. Therefore, it is imperative that sufficient numbers of women are included in all the phases of clinical trials.

**Fig. 1:**
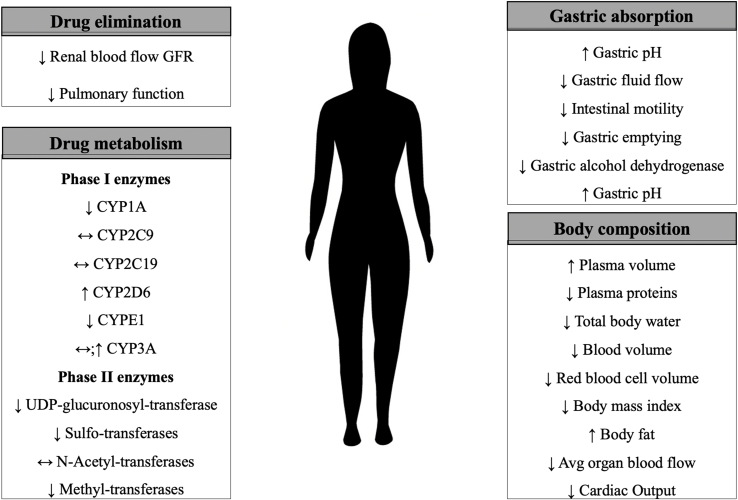
Pharmacokinetics/dynamics in women compared to men.

Several mechanisms influence sex differences in pharmacokinetics and pharmacodynamics in drug distribution and metabolism. Differences in drug distribution are affected by higher average percent body fat in women, the influence of sex hormones on binding proteins, lower glomerular filtration rates, and differences in blood flow and plasma volumes [1], [2], [3]. Pharmacogenetic factors also impact response to drug therapy [3]. Phase I and II enzymes have shown sex differences that could be affected by germline variants. These sex differences could lead to women exhibiting higher CYP2B6, CYP2D6, and CYP3A4 activity, whereas men exhibit higher CYP1A activity [4]. Differences in metabolism include sex differences in CYP450 enzymes and p-glycoprotein, which can affect the clearance of medications [1], [5]. Such differences can lead to higher or lower drug concentrations in women, affecting efficacy and adverse effects. For example, adverse effects of certain cardiovascular drugs have been reported more likely in women than men [1]. Women have a higher risk of drug-induced torsade de pointes (at least partially related to a longer QT interval among women), cough with ACE inhibitors, hemorrhagic complications with anticoagulants, electrolyte abnormalities with diuretics, and myopathy with statins [6]. Fig. 1 describes the parameters that influence drug distribution and metabolism that may be influenced by sex.

In 1993, the FDA established a committee to increase enrollment of women in clinical trials, and recently it established a website reporting “snapshots” highlighting differences among demographic groups and sex [7]. This initiative aggregated data from 292,766 clinical trial participants between 2015 and 2019 of FDA-approved medications. Across all FDA studies, women comprised 49 % of participants. However, women are only approximately one-third of trial participants in cardiovascular trials.

The participation prevalence ratio (PPR) is a metric recently described that accounts for the sex-specific prevalence of the condition being evaluated (with 0.8–1.2 considered adequate representation) [8]. The participation of women in CVD trials by PPR is very low: congestive heart failure is 0.5, acute coronary syndrome (CAD) 0.6, atrial fibrillation 0.8, and hypertension 0.9 [8]. One study compared sex-specific reporting of efficacy and safety outcomes for cardiovascular drug interventions presented at the major cardiology meetings (European Society of Cardiology, American Heart Association, American College of Cardiology) before and after the publication of the statements from these international cardiology societies emphasizing its importance [9]. There were 29 trials in 2010 and 34 in 2017. There were 32.8 % female participants in the 2010 studies, which only increased to 33.4 % in 2017. Particularly disheartening was that sex-specific reporting was 34.5 % in 2010 and declined to 23.5 % in 2017, with sex-specific safety outcomes reporting declining from 11.1 % to 8.6 %.

Sex-specific results are often provided in the form of subgroup analyses. Conclusions drawn from subgroup analysis are limited, and there is evidence that subgroup analysis is often poorly conducted and reported [10], [11], [12]. Some trials included a *p*-value for interaction when conducting sub-group analysis, which is important to understand a subgroup effect [13], [14]. However, many do not. Therefore, the inclusion of stratification by sex in a subgroup has limited utility in commenting on differences between the effects of drugs. There must be higher numbers of women involved in trials to improve these analyses.

**Fig. 2:**
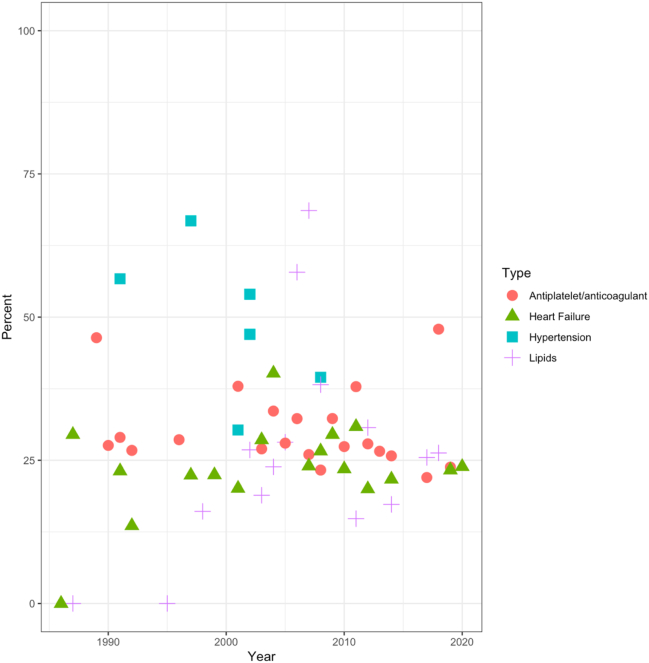
Scatterplot of the percentage of women in clinical trials by year Scatter plot of percent of women included in clinical trials versus year by drug category. When there was more than one trial in a given year, a weighted average was used. Overall, there was a minimal trend to increased participation of women. Most trials included <50 % of women. Seven out of 10 (70 %) blood pressure, 3 out of 22 (13.6 %) lipid, 13 out of 39 (33.3 %) antiplatelet/anticoagulation, and 14 out of 30 (46.7) heart failure trials did not include sex-stratified results.

This review evaluates female participation and sex-specific results in landmark drug trials in hypertension, lipid-lowering, anticoagulation/antiplatelets, and heart failure. A landmark trial was defined as an influential paper that has significantly impacted the knowledge and/or clinical practice as determined by the authors' opinions and consensus. A trial search was conducted via the MEDLINE database and Google Scholar using search terms “landmark trials” and “cardiology,” “heart failure,” “hypertension,” and “cardiovascular health.” A comparison of these landmark clinical trials with the percent of women included and sex-stratified results, if provided, is explored (Fig. 2).

## 2 Blood pressure-lowering therapy

Hypertension is a key contributor to the global CVD burden and can result in a wide variety of complications. Traditionally thought of as an issue more prevalent in post-menopausal women, data from the ARIC study indicates that it may be increasing in the younger female population [15]. Further, as women are delaying childbearing, hypertensive disorders of pregnancy are increasing in frequency [16]. Even though antihypertensive agents are beginning to be utilized more in pregnancy, there is still a paucity of clinical trial data in pregnant women, with most drugs having limited or conflicting data with recommendations to be used with caution (formerly FDA class C or B) [17], [18].

Data suggests that the female sex is likely a risk factor for hyponatremia and hypokalemia, leading to higher rates of hospitalizations in the prescription of diuretics like thiazides [19]. Additionally, pharmacogenomic factors can influence hydrochlorothiazide (HCTZ) response via a sex-specific interaction between insertion/deletion polymorphism in the ACE gene. Better BP response was noted in females with more insertion alleles than males with deletion alleles were associated with better BP response (*n* = 206 individuals included in the study) [20].

Sex-related differences in renin-angiotensin-aldosterone system (RAAS) activity are present secondary to hormonal regulation. Estrogens inhibit sympathetic nerve discharge, which leads to renin production. Men tend to have higher RAAS activity when compared to premenopausal women [3]. Higher peak concentrations were observed in women with commonly used angiotensin receptor blockers (ARBs). However, the differences were not significant when adjusted for weight except for peak serum concentration for telmisartan. This difference was attributed to a slower clearance. Further, there was no difference in blood pressure [21].

Landmark clinical trials in hypertension have included a range of female participants from 30 % to 67 % (Table 1). Interestingly, even early trials had a high percentage of women included. The SHEP trial in 1991 evaluated the impact of antihypertensive treatment with chlorthalidone ± atenolol on stroke risk and included 57 % women participants [22]. The Syst-Eur trial was a randomized controlled trial in 1997 and evaluated blood pressure treatment with nitrendipine, enalapril, and hydrochlorothiazide. This trial included an impressive 66.8 % women, yet sex-specific outcomes were not reported.

**Table 1:** Blood pressure.

Trial name, year	Total participants	Percent women participants	Patient population	Intervention, follow up	End point	Sex-specific result
SHEP, 1991	4736	56.7	Isolated systolic hypertension	Chlorthalidone ± atenolol ± reserpine vs placebo 4.5 years (mean)	Fatal and nonfatal stroke	Not provided
Syst-Eur, 1997	4695	66.8	Patients with hypertension	Nitrendipine ± enalapril ± hydrochlorothiazide vs placebo 2 years (median)	Death, stroke, retinal hemorrhage or exudates, myocardial infarction, congestive heart failure, dissecting aortic aneurysm, and renal insufficiency	Not provided
PROGRESS, 2001	6105	30.3	Non-hypertensive patients with a history of stroke or TIA	Perindopril ± indapamide 3.9 years (mean)	Total stroke (fatal or nonfatal)	Not provided
ALLHAT, 2002	33,357	47	Aged 55 years or older with hypertension and at least 1 other CHD risk factor	Chlorthalidone vs amlodipine vs lisinopril 4.9 years (mean)	Combined fatal CHD or nonfatal myocardial infarction	Amlodipine vs chlorthalidone relative risk (95 % confidence interval): Men: 0.98 (0.87–1.09) Women: 0.99 (0.85–1.15) Lisinopril vs chlorthalidone relative risk (95 % confidence interval): Men: 0.94 (0.85–1.05) Women: 1.06 (0.92–1.23)
LIFE, 2002	9193	54	Patients aged 55–80 with essential hypertension and LVH	Losartan vs atenolol 4.8 years (mean)	Composite endpoint of stroke, myocardial infarction, and cardiovascular death	Not provided
VALUE, 2004	15,245	Not provided	Outcomes in hypertensive patients at high cardiovascular risk treated with regimens based on valsartan or amlodipine: the VALUE randomized trial	Valsartan vs amlodipine 4.2 years (mean)	Time to first cardiac event (sudden cardiac death, fatal myocardial infarction, death due to heart failure, etc.)	Not provided
ACCOMPLISH, 2008	11,506	39.5	Patients with hypertension and at high risk for cardiovascular events	Benazepril + amlodipine vs benazepril + hydrochlorothiazide 36 months (mean)	Composite of death from cardiovascular causes, nonfatal myocardial infarction, nonfatal stroke, hospitalization for angina, resuscitation after sudden cardiac arrest, and coronary revascularization.	Benazepril-amlodipine vs benazepril-hydrochlorothiazide no. with primary end point (%), hazard ratio (95 % confidence interval): Men: 365 (10.6) vs 461 (13.1), 0.80 (0.69–0.91) Women: 187 (8.1) vs 218 (9.7), 0.83 (0.68–1.01)
CORAL, 2014	947	49.1	Patients with atherosclerotic renal-artery stenosis and hypertension	Medical therapy alone vs medical therapy plus renal artery stenting 43 months (median)	Composite end point of death from cardiovascular or renal causes, MI, stroke, hospitalization for CHF failure, progressive renal insufficiency, or need for renal replacement therapy	Stent + medical therapy vs medical therapy alone for primary endpoint (%) (hazard ratio; 95 % confidence interval): Men: 31.6 % vs 33.8 (0.89; 0.65–1.22) Women: 38.2 vs 37.8 (0.99; 0.74–1.33) *P* value for interaction 0.64
PATHWAY-2, 2015	335	31 %	Patients with resistant hypertension	Addition of spironolactone to a regimen 12 weeks	Systolic blood pressure reduction	Not provided
SPYRAL HTN-ON MED, 2018	80	84 %	Patients with resistant hypertension	Medical therapy alone vs medical therapy + renal denervation 6 months	Blood pressure change from baseline	Not provided

Abbreviations: please see abbreviation list of drug studies.

Despite relatively higher participation of women in antihypertensive trials, there are limited sex-specific results as most studies did not provide sex-stratified findings. A sub-analysis of the ALLHAT trial (which compared lisinopril vs. amlodipine vs. chlorthalidone) found that both sexes decreased blood pressure in all treatment groups. However, the decreases in systolic BP were slightly less in women compared to men. The percentage of participants with controlled BP (<140/90) was lower in women than in men by 1–6 %, varying by treatment group. However, there was no difference in cardiovascular outcomes [23]. The SPRINT trial (36 % women) found that intensive blood pressure control was associated with lower rates of major adverse cardiovascular events. Subgroup analysis found no heterogeneity of effect between men and women. Compared to the standard treatment group, the primary composite outcome in the intensive group was reduced by 16 % (HR 0.84 CI 0.61–1.13) in women and by 27 % in men (HR 0.73 CI 0.59–0.89) with a *p*-value for interaction 0.45 [24].

The ACCOMPLISH trial, which evaluated benazepril + amlodipine vs. benazepril + hydrochlorothiazide on a composite endpoint of cardiovascular events and death, demonstrated a slightly smaller absolute risk reduction in women (8.1 % vs. 9.7 %, hazard ratio 0.83 with confidence interval 0.68–1.01) versus men (10.6 % vs. 13.1 %, hazard ratio 0.80 with confidence interval 0.96–0.91) in the primary endpoint. However, there were only 39.5 % women participants [25].

In summary, there is evidence of sex-specific differences in the renin-angiotensin-aldosterone axis and response to medications secondary to both pharmacogenetics and hormones. Landmark clinical trials in hypertension have an overall higher percentage of women, even in early trials. However, there are limited sex-specific results.

## 3 Lipid-lowering therapy

Lipid-lowering therapy is a mainstay in the prevention and treatment of cardiovascular disease in both men and women. Drugs in this category include statins, fibrates, niacin, EPA/omega 3, ezetimibe, and PCSK9 inhibitors. Historically, the earliest lipid-lowering trials in the pre-statin era (before the 1990s) included men only, such as Coronary Drug Project [26], LRC-CPPT [27], and Helsinki Heart Study [28]. Female participation in lipid-lowering trials may have multiple contributing factors. As women develop cardiovascular disease later in life than men, trials that exclude older adults tend to recruit fewer female participants. This difference could be a critical factor in the lower enrollment of women in secondary prevention lipid therapy trials. For example, the PROSPER trial, which evaluated the benefits of pravastatin treatment specifically in an elderly population aged ≥70 years, successfully enrolled 52 % women [29].

Another obstacle could be higher statin intolerance, resulting in higher discontinuation of statin therapy by women than men [30]. Data also suggest a higher incidence of myopathy in women [31], [32].

Pharmacogenetics also plays a role in lipid metabolism and response to treatment. Peroxisome proliferator-activated receptor alpha (PPAR-alpha), the target of fibrates, regulates the expression of several genes in lipid metabolism. One study found that fenofibrate elevated the transcriptional activation of PPAR-alpha target genes in males greater than in females [33]. Other studies tested polymorphisms in the gene that encodes an estrogen receptor (ESR1) in patients treated with statins and observed sex-specific association with total cholesterol, HDL, and triglyceride levels [34], [35].

A systematic review of 60 lipid-lowering therapy randomized clinical trials published since 1990 demonstrated overall representation of women was 28.5 % (95 % CI 24.4 %–32.4 %), with a small increase in the proportion of women from 19.5 % to 33.6 % between 1990 and 2018 [36]. Only 53 % (95 % CI 41.8 %–65.3 %) of trials reported sex-specific outcomes, which did not increase over time significantly (p for trend 0.42), with statin trials having the highest reporting of outcomes according to sex (53 %; 95 % CI 36.4 %–69.1 %) [37].

Table 2 demonstrates a list of select landmark clinical trials from 1987 to 2018. Among these trials, the average participation of women was 27.3 %. As previously mentioned, several trials were men only, while some more recent trials had >50 % participation. For example, the JELIS trial, conducted in Japan, demonstrated a considerable reduction of CV events with eicosapentaenoic acid (EPA) treatment. This trial excluded premenopausal women and women older than 75 years; however, it still enrolled 68.6 % women despite those restrictions.

**Table 2:** Lipids.

Trial name, year	Total participants	Percent women participants	Patient population	Intervention, follow up	End point	Sex-specific result
Helsinki Heart Study, 1987	4081	0	Patients with primary dyslipidemia	Gemfibrozil vs placebo 60.4 months (mean)	Cardiac endpoints including myocardial infarction, sudden cardiac death, unwitnessed death	NA
WOSCOPS, 1995	6595	0	Men with elevated cholesterol level	Pravastatin vs placebo 4.9 years (mean)	Nonfatal myocardial infarction or death from coronary heart disease as a first event	NA
AFCAPS/TexCAPS, 1998	6605	15.1	Patients without coronary heart disease with average total cholesterol and below average HDL levels	Lovastatin vs placebo 5.2 years (mean)	First acute major coronary event defined as fatal or nonfatal myocardial infarction, unstable angina, or sudden cardiac death	Number of events lovastatin vs placebo: Men: 109 vs 170 Women: 7 vs 13
LIPID, 1998	9014	16.8	Patients with history of myocardial infarction or hospitalization for unstable angina and initial plasma total cholesterol levels 155–271 mg/dL	Pravastatin 40 mg vs placebo 6.1 years (mean)	Mortality from coronary heart disease	Percent reduction in risk (95 % confidence interval): Men: 26 (17–35) Women: 11 (−18–33)
HPS, 2002	20,536	24.7	Patients with coronary disease or diabetes	Simvastatin vs placebo 5 years (mean)	Mortality and fatal/nonfatal vascular events	Simvastatin vs placebo percent of events: Men: 21.6 vs 27.6 Women: 14.4 vs 17.7
PROSPER, 2002	8804	34.1	Patients with history or risk factors for vascular disease	Pravastatin vs placebo 3.2 years (mean)	Composite of coronary death, nonfatal myocardial infarction, and fatal or nonfatal stroke	Placebo vs pravastatin hazard ratio (95 % confidence interval): Men: 0.77 (0.65–0.92) Women: 0.96 (0.79–1.18)
ASCOT-LLA, 2003	19,342	18.9	Hypertensive patients with at least three other cardiovascular risk factors	Atorvastatin vs placebo 3.3 years (median)	Nonfatal myocardial infarction and fatal coronary heart disease	Atorvastatin vs placebo hazard ratio (95 % confidence interval): Men: 0.59 (0.44–0.77) Women: 1.10 (0.57–2.12)
CARDS, 2004	2838	32.1	Patients with type 2 diabetes without high LDL	Atorvastatin vs placebo 3.9 years (median)	Time to first occurrence of the following: acute coronary heart disease events, coronary revascularization, or stroke	Not provided
4S, 2004	4444	18.6	Patients with angina or prior myocardial infarction and serum cholesterol 5.5–8 mmol/L	Simvastatin vs placebo 5.4 years (median)	Mortality	Relative risk (95 % confidence interval) of death: Women: 1.12 (0.65–1.93) Men: 0.66 (0.53–0.80)
PROVE IT-TIMI 22, 2002	4162	21.9	Patients who had been hospitalized for acute coronary syndrome	Pravastatin 40 mg vs atorvastatin 80 mg 24 months (mean)	Composite of death from any cause, myocardial infarction, documented unstable angina requiring rehospitalization, revascularization (performed at least 30 days after randomization), and stroke	Atorvastatin vs pravastatin 2-year event rates (percent): Men: 23. vs 26.2 Women: 20.3 vs 27.0
TNT, 2005	10,001	19.2	Patients with clinically evident coronary heart disease and LDL <130 mg/dL	Atorvastatin 10 mg vs atorvastatin 80 mg 4.9 years (median)	Occurrence of a first major cardiovascular event, defined as death from CHD, nonfatal non–procedure-related myocardial infarction, resuscitation after cardiac arrest, or fatal or nonfatal stroke	Published later in secondary analysis Women: HR 0.73; 95 % CI 0.54–1.00, *p* = 0.049 Men: HR 0.79; 95 % CI 0.69–0.91, *p* = 0.001
FIELD, 2005	9795	37.3	Patients with type 2 diabetes mellites	Fenofibrate vs placebo 5 years	Coronary events (coronary heart disease death or nonfatal myocardial infarction)	Proportion of events (%) placebo vs fenofibrate: Men: 16.6 vs 15.4 Women: 9.5 vs 7.7
MEGA, 2006	7832	68.5	Patients with hypercholesterolemia and no history of coronary artery disease or stroke	Pravastatin + diet vs diet alone 5.3 years (mean)	First occurrence of coronary heart disease	Hazard ratio (95 % confidence interval): Men: 0.63 (0.42–0.95) Women: 0.71 (0.44–1.14)
SPRACL, 2006	4731	40.2	Patients with history of stroke or transient ischemic attack within 1 to 6 months before study entry, LDL levels 100–190 mg/dL, and no coronary heart disease	Atorvastatin 80 vs placebo 4.9 years (median)	First nonfatal or fatal stroke	Published later in secondary analysis Women: HR 0.84; 95 % CI 0.63–1.11 Men: HR 0.84; 95 % CI 0.68–1.02 *P*-value for treatment × sex interaction *P* = 0.99
JELIS, 2007	18,645	68.6	Patients with total cholesterol 6.5 mmol/L or greater	Eicosapentaenoic acid (EPA) + statin vs statin alone 4.6 years (mean)	Any major coronary event, including sudden cardiac death, fatal and nonfatal myocardial infarction, and other nonfatal events	Women: HR 0.87; 95 % CI 0.68–1.13 Men: HR 0.76; 95 % CI 0.62–0.94 *p* = 0.43 for difference by sex
JUPITER, 2008	17,802	38.2	Healthy patients with LDL < 130 mg/dL and high CRP levels	Rosuvastatin vs placebo 1.9 years (median)	Combined myocardial infarction, stroke, arterial revascularization, hospitalization for unstable angina, or death from cardiovascular causes.	Relative hazard reductions in rosuvastatin group: Men: 42 % Women: 46 %
ACCORD-Lipids, 2012	5518	30.7	Patients with type 2 diabetes mellites	Fenofibrate vs placebo 4.7 years (mean)	First occurrence of nonfatal myocardial infarction, nonfatal stroke, or death from cardiovascular causes	Fenofibrate vs placebo % of events: Men: 11.18 vs 13.30 Women: 9.05 vs 6.64
AIM-HIGH, 2011	3414	14.8	Patients with cardiovascular disease	Niacin vs placebo 3 years (mean)	Composite of death from coronary heart disease, nonfatal myocardial infarction, ischemic stroke, hospitalization for an acute coronary syndrome, or symptom-driven coronary or cerebral revascularization	Forest plot of men vs women hazard ratios
HPS2-THRIVE	25,673	17.3	Patients with atherosclerotic disease	Niacin + laropiprant vs placebo 3.9 years (median)	First major vascular event	Niacin vs placebo event rates: Men: 13.2 vs 14 Women: 13.4 vs 12.13
FOURIER, 2017	27,564	25.5	Patients with atherosclerotic cardiovascular disease and LDL > 70 mg/dL	Evolocumab vs placebo 2.2 years (median)	Composite of cardiovascular death, myocardial infarction, stroke, hospitalization for unstable angina, or coronary revascularization. The key secondary efficacy endpoint was the composite of cardiovascular death, myocardial infarction, or stroke	EvoMab vs Placebo percent event rate of primary endpoint, hazard ratio (confidence interval): Men: 10.3 vs 11.8 (0.86, 0.80–0.94) Women: 8.1 vs 9.9 (0.81 (0.69–0.95))
REDUCE-IT, 2018	8179	28.8	Patients with elevated triglycerides levels	Icosapent ethyl vs placebo 4.9 years (median)	Composite of cardiovascular death, nonfatal myocardial infarction, nonfatal stroke, coronary revascularization, or unstable angina	Icosapent ethyl vs placebo hazard ratio (95 % confidence interval): Men: 0.73 (0.65–0.82) Women: 0.82 (0.66–1.01)
ODYSSEY, 2018	18,924	25.2	Patients with acute coronary syndrome	Alirocumab vs placebo 2.8 years (median)	Composite of death from coronary heart disease, nonfatal myocardial infarction, fatal or nonfatal ischemic stroke, or unstable angina requiring hospitalization.	Alirocumab vs placebo hazard ratio (95 % confidence interval): Men: 0.83 (0.74–0.92) Women: 0.91 (0.77–1.08)

Abbreviations: please see abbreviation list of drug studies.

Some of the clinical trials demonstrated sex differences in their results. IMPROVE-IT (2017, 24 % women) showed that the addition of ezetimibe to simvastatin had a greater risk reduction in women (12 %) than men (5 %) for the primary composite endpoint [38]. Sex-specific data is variable among non-LDL-focused drug therapies, including niacin, fibric acid derivatives, and omega 3-fatty acids. For example, clinical trials on niacin included only a very small proportion of women, even in more recent trials like HPS2-THRIVE (17 % women) in 2014 and AIM-HIGH (<15 % women) in 2011 [39], [40]. Pre-specified analysis of HPS2-THRIVE based on sex showed a trend towards worse CV outcomes in women treated with niacin (*p* = 0.07).

The limited number of women or lack of reporting in primary prevention trials significantly limits sex-specific analysis. Therefore most sex-specific data on outcomes is derived from meta-analyses. Two primary prevention meta-analyses from 2004 and 2010 did not show significant CVD event reduction in women with statins [41], [42], [43]. On the contrary, in 2008, the landmark JUPITER trial was published (17,802 participants, 38 % women), demonstrating that rosuvastatin used for primary prevention reduced CV events in women and has a relative risk reduction similar to that in men [44]. In addition, further analysis of their data demonstrated that women had a significantly greater reduction compared to men in revascularization/unstable angina [45].

Overall, there are sex-specific differences in lipid metabolism, pharmacogenetics, and adverse medication effects. These adverse effects may play a role in limiting the participation of women in clinical trials. Many trials did not include sex-stratification in the initial study but later performed stratified analyses.

## 4 Antiplatelets and anticoagulation

Antiplatelet agents, including aspirin, ticagrelor, clopidogrel, and prasugrel, have consistently demonstrated benefits in CAD. Oral anticoagulation agents include warfarin, apixaban, and rivaroxaban and play an important treatment role in atrial fibrillation. However, the limited inclusion of women in cardiovascular trials has restricted the research on sex differences on the effect of these drugs.

Sex-related differences in both pharmacokinetics and pharmacodynamics are present in antiplatelet and anticoagulant therapies. Numerous contributing factors include a decreased volume of distribution, differing body composition, and metabolic effects driven by hormonal differences that change in various life stages [46]. Women tend to have higher platelet counts than men [47]. These differences lend to extended bleeding times in women [46]. Women taking warfarin experienced more minor bleeding complications than men and required less drug per week to maintain their International Normalized Ratio (INR) [48].

A comparison of 39 antiplatelet and anticoagulant landmark drug trials (including aspirin, clopidogrel, ticagrelor, prasugrel, warfarin, apixaban, and rivaroxaban) from 1988 to 2019 is shown in Table 3. The total number of participants was 447,496 across all trials, and the percentage of women included ranged from 0 % to 56.4 %. On average, the percentage of women included was 30.1. Only one trial, ASPREE (2018), had greater than half female participants. This trial evaluated aspirin versus placebo in patients >70 years of age without cardiovascular disease. This study provided sex-stratified results in a supplementary table and did not demonstrate a significant difference in results between men versus women.

**Table 3:** Antiplatelet/anticoagulants.

Trial name, year	Total participants	Percent women participants	Patient population	Intervention, Follow up	End point	Sex-specific result
ISIS-2, 1988	17,187	Not recorded	Patients with acute MI	Aspirin and streptokinase 15 months (median)	Vascular mortality at 5 weeks	Not reported
AFASAK, 1989	1007	46.4	Outpatients with chronic, non-rheumatic atrial fibrillation	Warfarin vs aspirin vs placebo 2 years	Thromboembolic complication	Not reported
BAATAF, 1990	420	27.6	Patients with non-rheumatic atrial fibrillation	Low-dose warfarin vs aspirin/placebo 2.2 years (mean)	Ischemic stroke	Not reported
SPAF, 1991	1330	29	Inpatient and outpatients with non-valvular atrial fibrillation	Aspirin vs warfarin vs placebo 1.3 years (mean)	Ischemic strokes and systemic emboli	Not reported
SPINAF, 1992	571	0	Male veterans with nonrheumatic atrial fibrillation	Low-dose warfarin vs placebo 1.7 years (mean)	Cerebral infarction	Not reported
ISIS-3, 1992	41,299	27.1	Patients with acute MI	Streptokinase, tPA, anistreplase plus aspirin and heparin or aspirin alone 6 months	Mortality	Not reported
SPAF-II, 1994	1100	Not reported	Patients with non-rheumatic atrial fibrillation	Aspirin vs warfarin 2 years (mean)	Ischemic strokes and systemic emboli	Not reported
CAPRIE, 1996	19,185	28	Patients with recent cardiovascular event (stroke or myocardial infarction)	Clopidogrel vs aspirin 1.91 years (mean)	Composite outcome cluster of ischemic stroke, myocardial infarction, or vascular death	Not reported
SPAF-III, 1996	1044	39.5	Patients with atrial fibrillation and at least one thromboembolic risk factor	Low dose, fixed-intensity warfarin and aspirin vs adjusted-dose warfarin 1.1 years (mean)	Ischemic strokes and systemic emboli	Not reported
ADMIRAL, 2001	300	18.3	Patients with acute MI	Abciximab plus stenting vs placebo vs stenting 30 days and 6 months	Composite of death, reinfarction, or urgent revascularization of the target vessel	Stent plus abciximab vs stent plus placebo 30 days RR (95 % CI): Men: 0.37 (0.15–0.87) Women: 1.38 (0.12–16.1) 6 months: Men: 0.40 (0.17–0.94) Women: 1.03 (0.16–6.69)
CURE, 2001	12,562	38.4	Patients with ACS without ST-segment elevation	Clopidogrel vs placebo 9 months (mean)	Composite of death from cardiovascular causes, nonfatal myocardial infarction, or stroke	Percent of patients with event in placebo vs clopidogrel: Men: 11.9 vs 9.1 Women: 10.7 vs 9.5
CADILLAC, 2003	2082	27	Patients with acute MI	Abciximab vs placebo 12 months	Composite endpoint of death, MI, ischemia-driven target-vessel revascularization (TVR), or disabling stroke at 30 days	Abciximab vs no abciximab relative risk (95 % CI): Men: 0.90 (0.71–1.15) Women: 0.96 (0.71–1.30)
SYNERGY, 2004	9978	33.6	Patients with NSTEMI ACS at high risk for ischemic cardiac complications	Enoxaparin vs unfractionated heparin 30 days	Composite clinical endpoint of all-cause death or nonfatal myocardial infarction during the first 30 days after randomization	Number (%) patients with death or MI at 30 days enoxaparin vs unfractionated heparin: Men: 467 (14.2) vs 506 (15.4) Women: 229 (13.5) vs 216 (12.9)
COMMIT, 2005	45,852	28	Patients admitted within 24 h of suspected acute MI onset	Clopidogrel plus aspirin vs placebo plus aspirin 28 days	Composite of death, reinfarction, or cardiac arrest; and death from any cause during the scheduled treatment period	Clopidogrel vs placebo number of events (%): Men: 1274 (7.7) vs 1416 (8.6) Women: 847 (13.3) vs 894 (14.0)
ACUITY, 2006	13,819	30	Patients with acute coronary syndrome undergoing invasive treatment	Bivalirudin vs heparin plus a glycoprotein IIb/IIIa inhibitor 1 year	Composite ischemia endpoint (death, myocardial infarction, or unplanned revascularization for ischemia)	Bivalirudin alone vs heparin plus IIb/IIIa inhibitor on composite ischemia relative risk (95 % CI): Men: 1.03 (0.87–1.22) Women: 1.23 (0.88–1.30)
OASIS-6, 2006	12,092	27.7	Patients with STEMI	Fondaparinux vs control 30 days–6 months	Composite of death or reinfarction at 30 days (primary) with secondary assessments at 9 days and at final follow-up (3 or 6 months)	Number (%) patients with event in Placebo vs Fondaparinux: Men: 390 (9.0) vs 339 (7.7) Women: 287 (16.9) vs 246 (15.0)
CHARISMA, 2006	15,603	29.8	Clinically evident cardiovascular disease or multiple risk factors	Clopidogrel plus aspirin vs placebo plus aspirin 28 months (median)	Composite of myocardial infarction, stroke, or death from cardiovascular causes	Hazard ratio forest plot with specific numbers for sex-stratified analysis provided
OASIS-5, 2006	20,078	38	Patients with ACS	Fondaparinux vs enoxaparin 90–180 days	Death, myocardial infarction, or refractory ischemia at nine days	Percentage of patients with an event taking enoxaparin vs fondaparinux: Men: 6.0 vs 5.8 Women: 5.3 vs 5.7
ACTIVE, 2006	6706	33.9	Patients with nonvalvular atrial fibrillation	Warfarin vs aspirin plus clopidogrel 1.28 years (median)	First occurrence of stroke, non-CNS systemic embolus, myocardial infarction, or vascular death	Not reported
TRITON-TIMI-38, 2007	13,608	26	Patients with moderate-to-high-risk ACS with scheduled PCI	Prasugrel vs clopidogrel 15 months (mean)	Death from cardiovascular causes, nonfatal myocardial infarction, or nonfatal stroke.	Prasugrel vs clopidogrel rates of primary endpoint: Men: 9.5 vs 11.9 Women: 11.0 vs 12.6
HORIZONS-AMI, 2008	3602	23.3	Patients with STEMI presenting within 12 h and undergoing primary PCI	Heparin plus glycoprotein IIb/IIIa inhibitor vs bivalirudin alone 30 days to yearly for 5 years	Combined adverse clinical events, defined as the combination of major bleeding or major adverse cardiovascular events, including death, reinfarction, target-vessel revascularization for ischemia, and stroke	Not reported
PLATO, 2009	18,624	28.4	Patients admitted to the hospital with ACS	Ticagrelor vs clopidogrel 12 months	Composite of death from vascular causes, myocardial infarction, or stroke	Ticagrelor vs clopidogrel hazard ratio (95 % CI): Men: 0.85 (0.76–0.95) Women: 0.83 (0.71–0.97)
PROTECT AF, 2009	707	29.7	Patients with non-valvular atrial fibrillation	Percutaneous closure of the left atrial appendage vs warfarin 18 months (mean)	Composite endpoint of stroke, cardiovascular death, and systemic embolism	Left atrial appendage closure vs warfarin hazard ratio (95 % confidence interval) Men: 0.32 (0.13–0.77) Women: 1.47 (0.52–4.11)
RE-LY, 2009	18,113	36.4	Patients with atrial fibrillation and a risk of stroke	Dabigatran vs adjusted-dose warfarin 2 years (median)	Stroke or systemic embolism	Dabigatran (110 mg) vs dabigatran (150 mg) vs warfarin %/year: Men: 1.35 vs 1.10 vs 1.49 Women: 1.86 vs 1.14 vs 2.03
CURRENT-OASIS-7, 2010	25,086	27.4	Patients with ACS referred for invasive strategy	Double vs single-dose clopidogrel, aspirin 30 days (mean)	Cardiovascular death, myocardial infarction, or stroke at 30 days	Double vs standard clopidogrel dose hazard ratio in men vs women: 1.00 vs 0.83 (*p* = 0.95 vs 0.09) Double vs standard aspirin hazard ratio in men vs women: 0.97 vs 0.97 (p = 0.95 vs 0.75)
ROCKET-AF, 2011	14,264	39.7	Patients with nonvalvular atrial fibrillation	Rivaroxaban vs dose-adjusted warfarin 707 days (median)	Stroke or systemic embolism	Rivaroxaban vs warfarin hazard ratio (95 % confidence interval): Men: 0.87 (0.7–1.09) Women: 0.89 (0.7–1.12)
ARISTOTLE, 2011	18,201	35.3	Patients with atrial fibrillation and at least one additional risk factor for stroke	Apixaban vs warfarin 1.8 years (mean)	Ischemic or hemorrhagic stroke or systemic embolism	Apixaban vs warfarin number of events (%/year): Male: 132 (1.2) vs 160 (1.5) Female: 80 (1.4) vs 105 (1.8)
AVERROES, 2011	5599	41.5	Patients with atrial fibrillation who were at increased risk for stroke and or whom vitamin K antagonist therapy was unsuitable	Apixaban vs aspirin 1.1 years (mean)	Stroke or systemic embolism	Aspirin vs apixaban number of events (%/year): Men: 49 (2.7) vs 26 (1.4) Women: 64 (4.9) vs 25. (1.9)
ATLAS-ACS-2-TIMIT-51, 2012	15,526	25.3	Patients with recent ACS	Rivaroxaban vs placebo 31 months (max)	Composite of death from cardiovascular causes, myocardial infarction, or stroke	Rivaroxaban vs placebo hazard ratio (95 % CI): Men: 0.87 (0.75–1.01) Women: 0.77 (0.60–0.99)
TRILOGY-ACS, 2012	7243	35.9	Patients with ACS treated with medical management without revascularization within 10 days of index event	Prasugrel vs clopidogrel 17 months (mean)	Composite of death from cardiovascular causes, nonfatal myocardial infarction, or nonfatal stroke	Prasugrel vs clopidogrel hazard ratio (95 % CI): Men: 0.86 (0.72–1.03) Women: 1.02 (0.80–1.29)
WARCEF, 2012	2305	20	Patients in sinus rhythm with reduced left ventricular ejection fraction	Warfarin vs aspirin 3.5 years (mean)	Time to first event in composite endpoint of ischemic stroke, intracerebral hemorrhage, or death from any cause	Not reported
WOEST, 2013	573	20.1	Patients undergoing percutaneous coronary intervention on oral anticoagulation	Clopidogrel vs clopidogrel plus aspirin 1 year	Any bleeding episode within 1 year of PCI	Triple vs double therapy number of events: Men: 234 vs 214 Women: 50 vs 65
ACCOAST, 2013	4033	27.5	Patients with NSTEMI ACS and positive troponin level scheduled to undergo coronary angiography	Prasugrel vs placebo before PCI 30 days	Composite of death from cardiovascular causes, myocardial infarction, stroke, urgent revascularization, or glycoprotein IIb/IIIa inhibitor rescue therapy (glycoprotein IIb/IIIa bailout)	Prasugrel vs placebo hazard ratio (95 % CI): Men: 0.99 (0.79–1.24) Women: 1.14 (0.76–1.70)
PRODIGY, 2014	9961	25.4	Patients having undergone coronary stent procedure with drug-eluting stent	Thienopyridine drug (clopidogrel or prasugrel) and aspirin for 12 months vs 30 months 30 months	Cumulative incidence of definite or probable stent thrombosis and major adverse cardiovascular and cerebrovascular events	Stent thrombosis continued thienopyridine vs placebo HR (95 % CI): Men: 0.21 (0.11–0.39) Women: 0.73 (0.28–1.19) Major adverse cardiovascular events thienopyridine vs placebo HR (95 % CI): Men: 0.69 (0.56–0.85) Women: 0.81 (0.56–1.17)
HEAT-PPCI, 2014	1829	27.8	Patients with STEMI undergoing PCI	Heparin vs bivalirudin 28 days	Composite of all-cause mortality, cerebrovascular accident, reinfarction, or unplanned target lesion revascularization	Not reported
COMPASS, 2017	27,395	22	Patients with stable atherosclerotic vascular disease	Rivaroxaban plus aspirin vs rivaroxaban vs aspirin 23 months (mean)	Composite of cardiovascular death, stroke, or myocardial infarction	Rivaroxaban plus aspirin vs aspirin hazard ratio (95 % confidence interval): Men: 0.76 (0.66–0.89) Women: 0.72 (0.54–0.97)
ASCEND, 2018	15,480	37.4	Patients with diabetes but no evident cardiovascular disease	Aspirin vs placebo 7.4 years (mean)	First serious vascular event (i.e., myocardial infarction, stroke or transient ischemic attack, or death from any vascular cause, excluding any confirmed intracranial hemorrhage)	Aspirin vs placebo HR (95 % confidence interval): Men: 0.86 (0.77–0.96) Women: 0.92 (0.78–1.09)
ASPREE, 2018	19,114	56.4	Patients 70 years old or greater without cardiovascular disease	Aspirin vs placebo 4.7 years (mean)	Composite of death, dementia, or persistent physical disability	Aspirin vs placebo HR (95 % confidence interval): Men: 0.99 (0.87–1.12) Women: 1.04 (0.91–1.18)
ISAR-REACT 5, 2019	4018	23.8	Patients with ACS with planned invasive strategy	Ticagrelor vs prasugrel 1 year	Composite of death, myocardial infarction, or stroke at 1 year	Ticagrelor vs prasugrel hazard ratio (95 % confidence interval) Men: 1.47 (1.13–1.90) Women: 1.10 (0.71–1.70)

Abbreviations: please see abbreviation list of drug studies.

Aspirin is a cornerstone of treatment for coronary artery disease. However, there is limited data studying its efficacy in women. The Women's Health Study consisted of 39,876 healthy women over 45 years, evaluating the use of aspirin in primary prevention of cardiovascular disease. It found a 17 % reduction in the risk of stroke but no significant effect on the risk of myocardial infarction or death from cardiovascular causes [49]. One meta-analysis of six trials published in 2006 found that in women, aspirin therapy was associated with a 12 % reduction in cardiovascular events and a 17 % reduction in stroke but no significant effect on myocardial infarction (MI).

In contrast, for men, aspirin was associated with a 14 % reduction in cardiovascular events and a 32 % reduction in MI but no significant effect on stroke [50]. This meta-analysis showed a similar risk of bleeding between the sexes. However, some studies report an increased risk of bleeding in women, at least in part due to excess dosing of drugs [51]. In another study evaluating 23 trials, authors found that trials that recruited women predominantly failed to demonstrate a significant risk reduction in nonfatal MI. In contrast, predominantly men trials demonstrated the largest risk reduction in nonfatal MI [52].

The DAPT trial investigated the benefits of 30 months vs. 12 months of dual antiplatelet therapy (DAPT) in patients receiving a drug-eluting stent. In the supplementary materials for that article, the authors report a subgroup analysis where there was definite or probable stent thrombosis in continued antiplatelet therapy versus placebo in 12 (0.3 %) vs. 55 (1.5 %) men and 7 (0.6 %) vs. 10 (0.8 %) women. The hazard ratio and 95 % confidence interval for men was 0.12 (0.11–0.39) versus 0.73 (0.28–1.91) in women. The calculated *p*-value for interaction was 0.04, an important measure of subgroup effect. However, only 25.4 % of the 9961 total participants in this study were female.

Sex differences do not seem to play a vital role in the de-escalation of DAPT. The TROPICAL-ACS trial assessed the impact of sex on clinical outcomes and found that the primary endpoint (combined ischemic and bleeding events) was not different [53]. A study in 2021 corroborated this, finding that while women had a higher bleeding risk than men, ischemic events were similar between sexes. The benefits of early aspirin withdrawal in patients after percutaneous coronary intervention (PCI) with continuing ticagrelor were comparable [54].

Out of 39 studies, 26 (66.7 %) published subgroup analysis, including effects stratified by sex. This information was usually available in a table in the text but only in supplementary materials in nine articles. Thirteen studies did not report sex-specific results, one of which had entirely male participants (Table 3).

In summary, there is evidence for sex differences in platelet and coagulation biology. However, clinical trials are quite limited in the inclusion of women. There is some evidence of a sex-based difference in bleeding risk but a general agreement of CV benefits in both women and men. However, most studies did not report sex stratification data.

## 5 Heart failure

The cornerstone for pharmacological treatment to reduce morbidity and mortality in patients with heart failure (HF) with a reduced ejection fraction (HFrEF) entails several key classes of medications, including angiotensin-converting enzyme (ACE) inhibitors, beta-blockers (BB), and mineralocorticoid receptor antagonists if the ejection fraction (EF) is ≤35 %. New therapies that have emerged include angiotensin receptor neprilysin inhibitors (ARNI) and sodium-glucose-linked transporter 2 inhibitors (SGLT2) [55]. Despite a similar lifetime risk of HF [56], female participation in clinical trials remains low. One barrier may be the higher proportion of trials targeted to HFrEF. In epidemiological studies, women with heart failure with preserved ejection fraction (HFpEF) outnumber men in a 2:1 ratio [56], [57]. The difference in incidence between men and women in HFpEF versus HFrEF is also represented in various disease registries. For example, of the 15,905 women enrolled in the Swedish Heart Failure Registry (n total = 42,987, 37 %), 55 % had HFpEF, 39 % had HF with mid-ranged EF, and 29 % had HFrEF [58]. In that registry, women comprised 55 % of all HFpEF diagnoses but only 29 % of HFrEF diagnoses. Unfortunately, a recent review noted that women's overall enrollment in HF trials has not increased over time [59].

Studies have demonstrated some sex differences in HF pharmacology. SGLT2 inhibitors are associated with higher rates of diabetic ketoacidosis and genitourinary infections in women [60], [61]. Mineralocorticoid receptor antagonists interact with estrogen signaling pathways and may have differential effects based on sex. One study in rats found that eplerenone attenuated LV chamber enlargement more effectively in females than in males and improved LVEF in females only. Additionally, the transcriptomic analysis revealed that in female rats, 19 % of downregulated genes and 44 % of upregulated genes after a myocardial infarction were restored to normal with eplerenone treatment versus only 4 % of genes restored in male rats [62]. Other classes of HF medications (i.e., BBs, ACE inhibitors) are discussed in the above section in hypertension.

Cytochrome 2D6 is a known genetic polymorphism, and one study evaluated CYP2D6 dependent beta-blocker (BB) (metoprolol, carvedilol, nebivolol, and propranolol) vs. independent BB (sotalol, bisoprolol, and atenolol). They noted increased adverse drug events in women compared to men for the CYP2D6 dependent BB vs. no difference in those that are independent [3], [63].

Table 4 demonstrates thirty landmark HF trials from 1986 to 2020 with varying degrees of women enrolled. The total number of participants was 118,282, and the average percent participation of women was 24.1 %. The modern era of HF trials began with the Captopril Multicenter Study, showing that endpoints such as exercise capacity and symptoms can be improved [64]. There were five female participants in this pivotal trial (*n* = 92). Later trials in the early 2000s had somewhat improved participation. For example, ValHeFT (evaluating the efficacy of valsartan) had 20.1 % female participation in 2001 and COMET (evaluating carvedilol versus metoprolol) had 20.5 % in 2003 [65]. However, the percentage of women included in trials seems to have relatively plateaued. The groundbreaking PARADIGM-HF trial in 2014 established that an ARNI was superior to enalapril in reducing the risks of death and hospitalization from heart failure [66]. Despite being a more recent clinical trial, there were only 21.7 % women.

**Table 4:** Heart failure.

Trial name, year	Total participants	Percent women participants	Patient population	Intervention, follow up	End point	Sex-specific result
V-HeFT, 1986	642	0	Chronic congestive heart failure	Prazosin vs Hydralazine-Isosorbide dinitrate 2.3 years (mean)	All-cause mortality	Not reported
CONSENSUS, 1987	253	29.5	NYHA class IV	Enalapril vs placebo 188 days (average)	Mortality	Not reported
SOLVD, 1991	6273	26.1	History of congestive heart failure and EF ≤	Enalapril vs placebo 1 year	Mortality	Not reported
V-HeFTII, 1991	804	0	NYHA class I-IV with EF ≤45 %	Enalapril vs Hydralazine-Isosorbide dinitrate 2.5 years (average)	All-cause mortality	Not reported
SOLVD-P, 1992	4228	11.5	Patients with EF ≤35 % not receiving drug treatment for heart failure	Enalapril vs placebo 37.4 months (mean)	Mortality	Not reported
SAVE, 1992	2231	17.5	Patients with EF ≤40 % after a myocardial infarction	Captopril vs placebo 42 months (mean)	Mortality	Risk Reduction 2 (95 % CI −53 to 37)
DIG, 1997	3397	22.4	NYHA class I-IV with ejection fraction ≤45 %	Digoxin vs placebo 37 months (mean)	All-cause mortality or all-cause admission	Not reported
RALES, 1999	1663	27	Severe Heart Failure and EF ≤ 35 %	Spironolactone vs placebo 24 months (mean)	All-cause mortality	Forest plot of men vs women hazard ratios
CIBIS-II, 1999	2647	19.5	NYHA class III, or IV with ejection fraction ≤35 %	Bisoprolol vs placebo 1.3 years (mean)	All-cause mortality	Not reported
MERIT-HF, 1999	3991	22.5	NYHA class II, III, or IV with ejection fraction ≤40 %	Metoprolol CR/XL vs placebo 1 year (mean)	All-cause mortality or all-cause admission	Forest plot of men vs women hazard ratios
ValHeFT, 2001	5010	20.1	NYHA class II, III, or IV heart failure	Valsartan vs placebo 23 months (median)	Mortality	Forest plot of men vs women hazard ratios
COPERNICUS, 2002	2289	Not recorded	NYHA class II, III, or IV and ejection fraction ≤ 25 %	Carvedilol vs placebo 10.4 months (mean)	All-cause mortality or all-cause admission	Forest plot of men vs women hazard ratios
CHARM-Added, 2003	2548	21.2	NYHA class II, III, or IV heart failure and EF ≤ 40 % being treated with ACE inhibitors	Candesartan vs placebo 41 months (median)	Death from cardiovascular disease or hospitalization for heart failure	Not reported
CHARM-Preserved, 2003	3023	40.1	NYHA class II, III, or IV and EF ≥ 40 %	Candesartan vs placebo 36.6 months (median)	Death from cardiovascular disease or hospitalization for heart failure	Not reported
CHARM-Alternative, 2003	2028	31.9	NYHA class II, III, or IV heart failure and EF ≤ 40 %	Candesartan vs placebo 33.7 months (median)	Death from cardiovascular disease or hospitalization for heart failure	Not reported
EPHESUS, 2003	6642	28.8	Clinical Heart Failure with EF ≤40 % after Myocardial Infarction	Eplerenone vs placebo, in addition to recommended therapy 16 months (mean)	Death from cardiovascular causes or hospitalization for heart failure, acute MI, stroke, or ventricular arrhythmia	Forest plot of men vs women hazard ratios
COMET, 2003	3029	20.5	NYHA class II, III, or IV with ejection fraction ≤35 %	Carvedilol 25 mg BID vs Metoprolol Tartrate 50 mg BID 58 months (mean)	All-cause mortality or all-cause admission	Hazard Ratio 0.97 (95 % CI 0.73–1.27)
A-HeFT, 2004	1050	40.2	NYHA class III, or IV with ejection fraction ≤45 %	Isosorbide-Dinitrate plus hydralazine vs placebo 10 months (mean)	All-cause mortality, first hospitalization for heart failure, and change in quality of life	Not reported
CORONA, 2007	5011	24	NYHA class II, III, or IV with ejection fraction ≤40 %	Rosuvastatin vs placebo 32.8 months (median)	Death from cardiovascular causes, nonfatal myocardial infarction, or nonfatal stroke	Placebo vs rosuvastatin rate: Men: 12.8 vs 12.0 Women: 10.8 vs 9.3
I-PRESERVE, 2008	4128	60.3	NYHA class II, III, or IV and EF ≥ 45 %	Irbesartan vs placebo 49.5 months (mean)	Death from cardiovascular disease or hospitalization for heart failure	Hazard Ratio 0.90 (95 % CI 0.78–1.05)
BEAUTIFUL, 2008	10,917	17	Stable CAD with ejection fraction <40 %	Ivabradine vs placebo 1.25 years (mean)	All-cause mortality or all-cause admission	Not reported
GISSI-HF, 2008	6975	21.7	NYHA class II, III, or IV	n-3 PUFA vs placebo 3.9 years (median)	Time to death, admission to hospital for cardiovascular reasons	Not reported
HEAAL, 2009	3846	29.5	NYHA class II, III, or IV heart failure and EF ≤ 40 % and intolerant of ACE inhibitors	Losartan 50 mg daily vs Losartan 150 mg daily 4.7 years (median)	Death from cardiovascular disease or hospitalization for heart failure	Hazard Ratio 1.02 (95 % CI 0.85–1.23)
SHIFT, 2010	6558	23.5	NYHA class I-IV and ejection fraction ≤ 35 %, sinus rhythm with heart rate >70 beats per min	Ivabradine vs placebo 22.9 months (median)	All-cause mortality or all-cause admission	Hazard Ratio 0.74 (95 % CI 06–0.91)
EMPHASIS-HF, 2011	2737	22.2	NYHA class II and EF ≤ 35 %	Eplerenone vs placebo, in addition to recommended therapy 22 months (median)	Death from cardiovascular disease or hospitalization for heart failure	Forest plot of men vs women hazard ratios
ASCEND-HF, 2011	7141	34.2	Admission with clinical heart failure with ejection fraction <40 %	Nesiritide vs placebo 30 days	Dyspnea, rehospitalization for heart failure, death within 30 days	Forest plot of men vs women hazard ratios
WARCEF, 2012	2305	20	Patients with reduced EF and in sinus rhythm	Warfarin vs aspirin 3.5 years (mean)	Ischemic stroke, intracerebral hemorrhage, or death from any cause	Not reported
PARADIGM-HG, 2014	8442	21.7	NHHA class II, III, or IV heart failure and an ejection fraction ≤ 40 %	LCZ696 vs enalapril in addition to recommended therapy 27 months (median)	Death from cardiovascular disease or hospitalization for heart failure	Forest plot of men vs women hazard ratios
TOPCAT, 2014	3445	51.5	HFpEF patients	Spironolactone vs placebo 3.3 years	Composite of death from cardiac arrest, heart failure hospitalization	Hazard ratio (95 % CI) Men: 0.89 (0.73–1.09) Women: 0.89 (0.71–1.12)
DAPA-HF, 2019	4744	23.3	NYHA class II, III, or IV heart failure and EF ≤ 40 %	Dapagliflozin vs placebo 18.2 (median)	Composite of worsening heart failure (hospitalization or urgent visit resulting in IV therapy for heart failure) or cardiovascular death	Hazard Ratio 0.79 (95 % CI of 0.59–1.04)
EMPEROR-Reduced, 2020	3730	23.9	NYHA class II, III, IV with an ejection fraction ≤40 %	Empagliflozin vs placebo 16 months (median)	Composite of cardiovascular death or hospitalization for worsening heart failure	Hazard Ratio 0.59 (95 % CI 0.44–0.08)

Abbreviations: please see abbreviation list of drug studies.

In a subgroup analysis of PARAGON-HF, women had a greater reduction in HF hospitalization [67]. In this study, for the primary outcome (hospitalizations for heart failure and death from cardiovascular causes), the rate ratio for sacubitril-valsartan versus valsartan was 0.73 (95 % CI, 0.59–0.90) in women and 1.03 (95 % CI, 0.84–1.25) in men (P interaction = 0.017). The difference was attributed to a reduction in HF hospitalization. The authors of that study do not propose a definite mechanistic basis for the noticed difference. However, they suggest it could be related to a higher normal left ventricular ejection fracture in women than in men or higher age-related arterial stiffening in women. Another possibility is the relationship between natriuretic peptides and sex hormones which may lead to lower levels of peptide levels in women after menopause [68].

The TOPCAT trial (52 % women) assessed the effect of spironolactone on a composite of cardiovascular death, cardiac arrest, or HF hospitalization in patients with HFpEF. While the primary analysis did not find that treatment had a significant outcome, an exploratory post-hoc analysis suggests possible sex differences in response. This analysis found no sex differences in the placebo or response arm outcomes for the primary outcome or its components. However, spironolactone was associated with reduced all-cause mortality in women (HR 0.66, *p* = 01) but not in men (*p*-value interaction = 0.02) [69]. I-PRESERVE was another trial in HFpEF (investigated the role of irbesartan) and had a majority of female participation (60.3 %) [70].

In summary, HFrEF has a greater prevalence in men and HFpEF in women. There is evidence of sex differences in medication effects, which could be hormonal or pharmacogenetic, but the underlying mechanism is unclear. Almost half (*n* = 14) of the trials did not provide sex-stratified results. Some studies include only a graphic of a forest plot representing hazard ratios and confidence intervals without providing specific numbers, case rates, or percentages. Overall, the representation of women in HF clinical trials is low and has not increased significantly over time.

## 6 Discussion

Cardiovascular drug trials have under-enrolled women historically compared to the prevalence of cardiovascular disorders according to sex. Participation varies by disease area, with the most underrepresented areas being HF, CAD, and acute coronary syndrome. The reasons for lack of inclusion are multifactorial. In HF, differences in prevalence in HFrEF vs. HFpEF in men and women may play a role. The under-enrollment of women in CAD may be related to the varied presentation of ACS in women and a lower likelihood of angiography in women, especially when used as inclusion criteria. Additionally, women tend to have less coronary artery disease and more ischemia without obstructive coronary arteries.

Age additionally may play a role. As women develop cardiovascular disease later in life than men, fewer female participants were recruited in trials that excluded older adults. One study found that CV trials were particularly low when the average participant age was 61–65 years [71]. Further, barriers like ageism may reduce the rate at which older women are referred to specialists like a cardiologist [72].

Pregnant women and women of childbearing potential are frequently excluded from clinical research. This exclusion limits the number of women eligible for any given research study, and it also limits research into the effect of medications in pregnancy.

There are several sex-specific differences in drug metabolism and efficacy, and it is imperative to ensure adequate representation of both men and women when evaluating these drugs. Recent studies have addressed why these gaps may exist and how to improve participation in the future. This change will require funding to power studies to look at sex-specific outcomes, which would significantly impact the population to enroll. Further, this will necessitate investment in an infrastructure that improves patient and provider awareness and knowledge, expanding access to trial design, and adequate representation in trial leadership. Currently, the FDA has no legal requirement for trials to have a certain percentage of patients in the subgroups. The FDA could consider including gender parity in enrollment or possibly target enrollment of women equal to the composition of the disease in the population (PPR of 1).

It is important to clarify the difference between sex and gender when writing in this space. Sex refers to the biological and anatomic categorization of males and females. Gender refers to the socially constructed norms that impose and influence the way individuals interact with the world [73]. This review focuses on sex or biological differences in cardiovascular pharmacology and drug effects. However, the effect of gender, namely the constraints historically and presently faced by women in our society, underpins the disparity in representation in clinical trials. Moreover, we recognize that other gender minorities, including transgender and nonbinary people, are not only underrepresented in clinical trials but that many clinical trials are limited in their reporting and inclusion of only the gender binary [74].

Moreover, there is a distinct racial disparity among the women included in clinical trials. The vast majority of female cardiology clinical trial participants were white. In the FDA Snapshots in cardiology trials, among women greater or equal to 65 years, 84 % were White, 10 % Asian, 2 % Black, 4 % other, and <1 % American Indian. Among women <65, 73 % were White, 16 % Asian, 6 % Black, 5 % other, and <1 % American Indian [7]. Barriers to representation are multifactorial and include community mistrust, transportation barriers, socioeconomic factors, lack of diversity in clinical trial leadership, inadequate outreach, and racism [75]. It is critically important that gender parity be paired with racial parity in representation in drug trials.

## 7 Conclusion

In the future, a collaboration between clinicians, scientists, patient advocacy, government, and industry will be required to develop an infrastructure and process to expand the participation of women in cardiovascular drug trials.

## Disclosures, author contributions, and funding

The authors have no conflicts of interest to disclose pertinent to this paper. There was no funding support provided for the manuscript preparation. The manuscript was divided into sections and equally written by all authors. The first author completed the primary manuscript review, and the senior author contributed to the concept, design, preparation, and content review of the final manuscript.

## Declaration of competing interest

The authors declare that they have no known competing financial interests or personal relationships that could have appeared to influence the work reported in this paper.

## Drug studies abbreviations

SHEP: Systolic Hypertension in the Elderly Program
Syst-Eur: Systolic Hypertension in Europe
PROGRESS: Preventing Strokes by Lowering Blood Pressure in Patients with Cerebral Ischemia
ALLHAT: Antihypertensive and Lipid-Lowering Treatment to Prevent Heart Attack Trial
LIFE: Losartan Intervention for Endpoint reduction in hypertension study
VALUE: Valsartan Antihypertensive Long-Term Use Evaluation
ACCOMPLISH: Avoiding Cardiovascular Events through Combination Therapy in Patients Living with Systolic Hypertension
CORAL: Cardiovascular Outcomes in Renal Atherosclerotic Lesions
PATHWAY-2: Prevention and Treatment of Hypertension With Algorithm-based therapy
SPYRAL HTN-ON MED: Global Clinical Study of Renal Denervation with the Symplicity Spyra Multi-electrode Renal Denervation System in Patients With Uncontrolled Hypertension on Standard Medical Therapy
WOSCOPS: West of Scotland Coronary Prevention Study
AFCAPS/TextCAPS: The Air Force/Texas Coronary Atherosclerosis Prevention Study
LIPID: Long-Term Intervention with Pravastatin in Ischaemic Disease
HPS: Heart Protection Study
PROSPER: Prospective Study of Pravastatin in the Elderly at Risk
ASCOT-LLA: Anglo-Scandinavian Cardiac Outcomes Trial--Lipid Lowering Arm
CARDS: Collaborative AtoRvastatin Diabetes Study
4S: Scandinavian Simvastatin Survival Study
PROVE IT-TIMI: Pravastatin or Atorvastatin Evaluation and Infection Therapy–Thrombolysis in Myocardial Infarction 22
TNT: Treating to New Targets
FIELD: Fenofibrate Intervention and Event Lowering in Diabetes
MEGA: Primary prevention of cardiovascular disease with pravastatin in Japan
SPRACL: Stroke Prevention by Aggressive Reduction of Cholesterol Levels
JELIS: Japan EPA lipid intervention study
JUPITER: Justification for the Use of Statins in Primary Prevention: An Intervention Trial Evaluating Rosuvastatin
ACCORD-Lipids: Action to Control Cardiovascular Risk in Diabetes – Lipids
AIM-HIGH: Atherothrombosis Intervention in Metabolic syndrome with low HDL/high triglycerides
HPS2-THRIVE: Heart Protection Study 2-Treatment of HDL to Reduce the Incidence of Vascular Events
FOURIER: Further Cardiovascular Outcomes Research with PCSK9 Inhibition in Subjects with Elevated Risk
REDUCE-IT: Reduction of Cardiovascular Events with Icosapent Ethyl–Intervention Trial
ODYSSEY: Long-term Safety and Tolerability of Alirocumab in High Cardiovascular Risk Patients with Hypercholesterolemia Not Adequately Controlled with Their Lipid Modifying Therapy
ISIS-2: International Study of Infarct Survival 2
AFASAK: Atrial Fibrillation, Aspirin, Antikoagulation
BAATAF: Boston Area Anticoagulation Trial for Atrial Fibrillation
SPAF: Stroke Prevention in Atrial Fibrillation Study
SPINAF: Stroke Prevention in Nonrheumatic Atrial Fibrillation
ISIS-3: International Study of Infarct Survival-3
SPAF-II: Stroke Prevention in Atrial Fibrillation Study II
ADMIRAL: Abciximab Before Direct Angioplasty and Stenting in Myocardial Infarction Regarding Acute and Long-term Follow-up
CURE: Clopidogrel in Unstable Angina to Prevent Recurrent Ischemic Events
CADILLAC: Controlled Abciximab and Device Investigation to Lower Late Angioplasty Complications
SYNERGY: Superior Yield of The New Strategy of Enoxaparin, Revascularization, And Glycoprotein IIb/IIia Inhibitors
COMMIT: Clopidogrel and Metoprolol in Myocardial Infarction Trial/Second Chinese Cardiac Study
ACUITY: Acute Catheterization and Urgent Intervention Triage Strategy Trial
OASIS-6: Organization for The Assessment of Strategies for Ischemic Syndromes 6
CHARISMA: Clopidogrel for High Atherothrombotic Risk, Ischemic Stabilization, Management, And Avoidance
OASIS-5: Comparison of Fondaparinux and Enoxaparin in Acute Coronary Syndromes
ACTIVE: Atrial Fibrillation Clopidogrel Trial with Irbesartan for Prevention of Vascular Events
TRITON-TIMI: Trial to Assess Improvement in Therapeutic Outcomes by Optimizing Platelet Inhibition With Prasugrel
HORIZONS-AMI: Harmonizing Outcomes with Revascularization and Stents in Acute Myocardial Infarction
PLATO: Platelet Inhibition and Patient Outcomes
PROTECT AF: Randomized Prospective Trial of Percutaneous Left Atrial Appendage Closure Versus Warfarin for Stroke Prevention in Atrial Fibrillation
RE-LY: Randomized Evaluation of Long-Term Anticoagulation Therapy
CURRENT-OASIS: Clopidogrel and Aspirin Optimal Dose Usage to Reduce Recurrent Events−Seventh Organization to Assess Strategies in Ischemic Syndromes
ROCKET-AF: Rivaroxaban Once Daily Oral Direct Factor Xa Inhibition Compared with Vitamin K Antagonism for Prevention of Stroke and Embolism Trial in Atrial Fibrillation
ARISTOTAL: Apixaban for Reduction in Stroke and Other Thromboembolic Events in Atrial Fibrillation
AVERROES: Apixaban Versus Acetylsalicylic Acid to Prevent Stroke in Atrial Fibrillation Patients Who Have Failed or Are Unsuitable for Vitamin K Antagonist Treatment
ATLAS-ACS-2 – TIMI 51: Anti-Xa Therapy to Lower Cardiovascular Events in Addition to Standard Therapy in Subjects with Acute Coronary Syndrome–Thrombolysis in Myocardial Infarction
TRILOGY-ACS: Targeted Platelet Inhibition to Clarify the Optimal Strategy to Medically Manage Acute Coronary Syndromes
WARCEF: Warfarin Versus Aspirin in Reduced Cardiac Ejection Fraction
WOEST: What is the Optimal antiplatElet and anticoagulant therapy in patients with oral anticoagulation and coronary StenTing
ACCOAST: A Comparison of Prasugrel at PCI or Time of Diagnosis of Non-ST Elevation Myocardial Infarction
PRODIGY: Prolonging Dual-Antiplatelet Treatment After Grading Stent-Induced Intimal Hyperplasia
HEAT-PPCI: How Effective are Antithrombotic Therapies in Primary Percutaneous Coronary Intervention
COMPASS: Cardiovascular Outcomes for People Using Anticoagulation Strategies
ASCEND: A Study of Cardiovascular Events in Diabetes
ASPREE: Aspirin in Reducing Events in the Elderly
ISAR-REACT: Intracoronary Stenting and Antithrombotic Regimen: Rapid Early Action for Coronary Treatment
